# CRISPR/Cas9-Engineered HEK293T Cellular Model Harboring the Pathogenic CDKL5 c.172A>T Variant Recapitulates Core Molecular Phenotypes of CDKL5 Deficiency Disorder

**DOI:** 10.5812/ijpr-172311

**Published:** 2026-06-13

**Authors:** Fatemeh Faghihi, Jafar Amani, Milad Gholami, Iman Salahshourifar

**Affiliations:** 1 Department of Biology, SR.C., Islamic Azad University, Tehran, Iran; 2 Applied Microbiology Research Center, Biomedicine Technologies Institute, Baqiyatallah University of Medical Sciences, Tehran, Iran; 3 Department of Biochemistry and Genetics, School of Medicine, Arak University of Medical Sciences, Arak, Iran

**Keywords:** CRISPR/Cas9, CDKL5, Autism Spectrum Disorder, Gene Editing, Cellular Model, Apoptosis

## Abstract

**Background:**

Mutations in the CDKL5 gene are strongly associated with severe neurodevelopmental disorders, including atypical Rett syndrome and autism spectrum disorder (ASD).

**Objectives:**

This study aimed to establish a cell model carrying a targeted CDKL5 mutation using the clustered regularly interspaced short palindromic repeats (CRISPR)/CRISPR-associated protein 9 (Cas9) system and to evaluate the phenotypic and molecular consequences.

**Methods:**

A single-guide RNA (sgRNA) targeting exon 2 of CDKL5 was designed using in silico tools to minimize off-target activity. The sgRNA and Cas9 nuclease were cloned into the pSpCas9(BB)-2A-green fluorescent protein (GFP) (PX458) vector and transfected into human embryonic kidney 293T (HEK293T) cells. Following fluorescence-activated cell sorting (FACS), genome-editing efficiency was quantified using a T7 endonuclease I (T7E1) assay and Sanger sequencing. Homology-directed repair (HDR) mediated by single-stranded oligodeoxynucleotide (ssODN) templates was used to introduce a specific missense mutation (c.172A>T; p.Lys58Met). Edited clones were evaluated for CDKL5 expression using reverse transcription-quantitative polymerase chain reaction (RT-qPCR), and cell survival and apoptosis were assessed using Annexin V/propidium iodide (PI) staining and caspase-3 activity assays.

**Results:**

The CRISPR/Cas9 construct achieved a mean on-target editing efficiency of 41.6 ± 3.2%, as determined by T7E1 digestion, and sequencing confirmed a heterozygous insertion in 28% of sorted clones. HDR-mediated precise editing was detected in 12% of clones. RT-qPCR analysis showed a 55% reduction in CDKL5 mRNA levels (P < 0.01) in edited cells, whereas Western blotting demonstrated a corresponding 48% decrease in protein expression. Functionally, mutant cells exhibited 25% lower viability (P < 0.05) and 2.3-fold higher apoptosis rates (P < 0.01) than wild-type controls. No significant off-target mutations were detected at the top five predicted loci.

**Conclusions:**

We successfully generated a human cell line model of CDKL5-related ASD using CRISPR/Cas9 technology. The mutation induced marked reductions in CDKL5 expression and cell survival. Although this HEK293T-based model provides a useful platform for mechanistic and screening studies, its non-neuronal origin limits its ability to fully recapitulate the neuronal context of CDKL5 deficiency disorder (CDD). This study establishes a novel, precisely engineered human cellular model using advanced CRISPR/Cas9 technology to mechanistically dissect the consequences of CDKL5 deficiency. By introducing a defined missense mutation (c.172A>T; p.Lys58Met) into HEK293T cells, this study provides a robust platform for investigating the role of CDKL5 in neurodevelopmental disorders and supports future therapeutic development.

## 1. Background

Autism spectrum disorder (ASD) comprises a heterogeneous group of neurodevelopmental conditions characterized by persistent deficits in social communication, restricted interests, and repetitive behaviors that emerge during early childhood. Over the past two decades, extensive epidemiological and molecular studies have shown that ASD is among the most genetically complex and heritable psychiatric disorders, with heritability estimates ranging from 60% to 90% (1, 2). Global prevalence has increased steadily and is now estimated at approximately 1 in every 100 children. Despite increasing awareness, the molecular mechanisms linking genetic mutations to the neurological and behavioral phenotypes of ASD remain only partially understood (3). Genetic studies have identified hundreds of candidate genes implicated in ASD, many of which encode synaptic scaffolding proteins, transcriptional regulators, and signaling molecules essential for brain development (4). A notable subset of these genes, including MECP2, FMR1, SHANK3, SCN2A, and CDKL5, participates in pathways that regulate synapse formation, dendritic spine morphology, and neurotransmission (5). Mutations in these genes often result in overlapping phenotypes, including intellectual disability, seizures, and impaired social cognition, underscoring convergent neurobiological mechanisms across syndromic and idiopathic ASD (6, 7). Among these genes, cyclin-dependent kinase-like 5 (CDKL5) has emerged as a particularly important gene in early-onset epileptic encephalopathies and atypical Rett syndrome (8). CDKL5 encodes a serine/threonine kinase that is highly expressed in excitatory cortical neurons and plays pivotal roles in dendritic arborization, axonal elongation, and synaptic plasticity (9). The gene is located on the X chromosome (Xp22), making males more vulnerable to homozygous loss-of-function mutations, which frequently result in lethality, whereas heterozygous females manifest variable neurological impairment due to X-inactivation mosaicism (10). Despite the critical role of CDKL5, developing precise human cellular models that enable direct investigation of specific mutations, particularly missense variants, has remained challenging. Existing models, such as those derived from patient fibroblasts or induced pluripotent stem cell (iPSC) lines, often have confounding genetic backgrounds or do not allow precise targeting of specific pathogenic alterations. This study addresses this gap by using CRISPR/Cas9 technology to create an isogenic HEK293T cell line with a defined pathogenic missense mutation (c.172A>T; p.Lys58Met), enabling focused examination of its direct molecular and cellular consequences.

The CDKL5 protein functions in both the cytoplasm and the nucleus, where it phosphorylates downstream substrates, including microtubule-associated proteins, MeCP2, and transcriptional coregulators (11). Disruption of CDKL5 kinase activity has been linked to altered microtubule dynamics, impaired axonal transport, and reduced dendritic spine density (12). In mouse models, Cdkl5 knockout leads to defective hippocampal long-term potentiation, learning and memory deficits, and increased neuronal apoptosis. Clinically, CDD presents with refractory seizures in infancy, severe motor impairment, and autistic-like behaviors (13, 14).

The advent of CRISPR/Cas9 endonuclease technology has revolutionized genome editing in eukaryotic systems. Derived from the adaptive immune system of bacteria and archaea, CRISPR/Cas9 uses an sgRNA to direct Cas9 to a complementary DNA sequence adjacent to a protospacer-adjacent motif (PAM), where it introduces a double-stranded break (15). This break is repaired either through the error-prone non-homologous end-joining (NHEJ) pathway, generating small insertions or deletions (indels), or through HDR in the presence of a donor template, enabling precise sequence modification (16, 17). While iPSC-derived neuronal models are invaluable for studying late-stage neuronal phenotypes, their derivation is time-consuming and can introduce variability. For initial mechanistic studies and high-throughput screening, engineered cell lines such as HEK293T cells offer distinct advantages, including rapid generation, ease of genetic manipulation, and a stable genetic background essential for isolating the direct effects of specific mutations. The strategic use of HEK293T cells, combined with precise HDR-mediated editing, allows robust assessment of the pathogenic effects of the CDKL5 c.172A>T (p.Lys58Met) variant. Owing to its simplicity, versatility, and cost-effectiveness, CRISPR/Cas9 has become the preferred strategy for functional genomics, disease modeling, and therapeutic gene-correction applications. In neuroscience, this technology has been successfully applied to manipulate ASD-related genes, including SHANK3, CNTNAP2, and MECP2, in cell lines and iPSCs (18, 19). Given the prominent involvement of CDKL5 in early cortical development and the limitations of animal models, CRISPR/Cas9-based human cell models offer a unique opportunity to explore the molecular pathology of CDKL5-related ASD. Previous studies using patient-derived fibroblasts and iPSC lines have demonstrated mitochondrial dysfunction, impaired autophagy, and reduced neuronal survival associated with CDKL5 mutations (20). However, targeted isogenic systems in which only CDKL5 is mutated in an otherwise identical genetic background are essential for dissecting direct causal effects (21).

The c.172A>T (p.Lys58Met) missense variant has previously been identified in male patients with early-onset epileptic encephalopathy and autistic features, highlighting its direct relevance to the spectrum of CDKL5 deficiency disorders, including ASD. Structural modeling predicts that this substitution disrupts a conserved catalytic loop within the N-terminal kinase domain, thereby impairing ATP binding and substrate phosphorylation, which are essential for the role of CDKL5 in dendritic spine maturation and synaptic signaling (22, 23).

## 2. Objectives

In this study, we used CRISPR/Cas9 to introduce a specific missense mutation (c.172A>T; p.Lys58Met) into exon 2 of the CDKL5 gene in HEK293T cells. This mutation was selected based on its predicted pathogenic impact on kinase-domain function and its association with ASD phenotypes. We also used ssODN-mediated HDR to achieve precise genome editing. Using integrated molecular and cellular assays, we characterized the effects of CDKL5 disruption on gene expression, protein levels, and cell viability.

## 3. Methods

### 3.1. Study Design and Overview

The primary objective of this study was to generate a human cell model harboring a pathogenic CDKL5 mutation using the CRISPR/Cas9 system and to evaluate its molecular and cellular phenotypes. All laboratory procedures were performed according to biosafety level II standards, and reagents were obtained from certified commercial suppliers.

The study consisted of three main phases: 1) design and cloning of CRISPR/Cas9 constructs targeting CDKL5, 2) generation and selection of edited HEK293T cell lines via HDR-mediated repair, and 3) molecular and cellular characterization of mutant clones.

### 3.2. Cell Line and Culture Conditions

Human embryonic kidney 293T cells (HEK293T; ATCC CRL-11268) were used because of their high transfection efficiency and suitability for stable genome editing. Cells were maintained in Dulbecco modified Eagle medium (DMEM; Gibco, USA) supplemented with 10% fetal bovine serum (FBS), 1% penicillin-streptomycin, and 2 mM L-glutamine at 37°C in a humidified incubator containing 5% CO_2_. Cells were passaged every 2 - 3 days using 0.25% trypsin-ethylenediaminetetraacetic acid (EDTA).

### 3.3. CRISPR/Cas9 Vector Construction

A single-guide RNA sequence targeting exon 2 of the CDKL5 gene was designed using the online Synthego CRISPR Design Tool (https://www.synthego.com) to ensure high on-target activity and minimal off-target potential. The sgRNA target sequence was as follows: forward, 5′-GGAGTTTGTCTTCATGAAGA-3′; reverse complement, 5′-TCTTCATGAAGACAAACTCC-3′. This sequence was located upstream of the PAM motif (5′-NGG-3′).

Annealed oligonucleotides were cloned into the pSpCas9(BB)-2A-GFP (PX458) vector (Addgene plasmid #48138) through BbsI restriction sites using a single-step digestion-ligation reaction consisting of 1 μg plasmid DNA, 1 μL BbsI enzyme, 0.5 μL T4 ligase, and 1 μL annealed oligonucleotides. Ligated products were transformed into *Escherichia coli* DH5α competent cells and plated on Luria-Bertani agar containing ampicillin (100 μg/mL). Positive colonies were confirmed by colony polymerase chain reaction (PCR) and Sanger sequencing using U6 promoter primers.

### 3.4. Homology-Directed Repair Template Design

To introduce the precise missense mutation (c.172A>T; p.Lys58Met), an ssODN was synthesized as the donor repair template. The 95-nucleotide ssODN contained 47-bp homology arms flanking the mutation site, with a silent PAM-site modification to prevent re-cutting:

5′-GTGGCTTGCATCAAAAGAGGAGTTTGTCTTCATGAAGATTCC TAACATTGGTAATGTGATGAATAAATTTGAGATCCTTGGGGTT GTAGGTGAAG-3′

### 3.5. Transfection and Fluorescence-Based Selection

HEK293T cells were seeded at a density of 2 × 10^5^ cells per well in 6-well plates 1 day before transfection. When cells reached approximately 70% - 80% confluence, they were cotransfected with 2.5 μg PX458-sgRNA plasmid and 50 pmol ssODN using 6.25 μL TransfectiMine reagent (Dara Zist Fan, Iran). Thus, the DNA:ssODN:TransfectiMine input amounts were 2.5 μg:50 pmol:6.25 μL per well. Briefly, plasmid DNA and ssODN were diluted together in 250 μL serum-free DMEM, whereas 6.25 μL TransfectiMine was diluted separately in 250 μL serum-free DMEM. The 2 solutions were gently mixed and incubated for 5 minutes at room temperature to allow complex formation. The resulting transfection mixture, with a final volume of 500 μL per well, was added dropwise to the cells. Six hours after transfection, the culture medium was completely removed and replaced with fresh complete DMEM supplemented with 5% FBS and penicillin-streptomycin.

Forty-eight hours after transfection, GFP-positive cells were isolated under sterile conditions using a BD FACSAria III flow cytometer (BD Biosciences, USA). Approximately 50% of the total cell population exhibited GFP fluorescence, indicating successful transfection. The sorted GFP-positive cells were subsequently expanded for 10 - 14 days in complete DMEM before downstream molecular analyses.

After expansion, genomic DNA was isolated from individual clones derived from the sorted GFP-positive cell population. Amplification refractory mutation system PCR (ARMS-PCR) was performed as an initial genotyping screen to identify candidate edited clones, after which a subset of 20 clones was randomly selected for Sanger sequencing to validate the genotype and classify editing outcomes as HDR-mediated substitutions, indel-containing clones, or wild-type clones.

### 3.6. Genomic DNA Extraction and Mutation Analysis

Genomic DNA was extracted using the SinaClon DNA Extraction Kit (Iran) according to the manufacturer’s protocol. Allele-specific mutation screening was performed using ARMS-PCR as an initial genotyping assay to identify candidate edited clones. This method used allele-specific primers whose 3′ terminal nucleotide was complementary to either the wild-type or mutant sequence. Because DNA polymerase cannot efficiently extend primers with a mismatched 3′ base, amplification occurred only when perfect complementarity was present, allowing discrimination of the c.172A>T substitution from the wild-type allele.

The target region encompassing exon 2 of CDKL5 was PCR-amplified using the following primers: forward, 5′-AAGTGCTGGCTAGTCTTCCG-3′; reverse, 5′-TGCTTCCCATCTTGACTGGA-3′. The PCR products were analyzed by 1.5% agarose gel electrophoresis and purified using a QIAquick Gel Extraction Kit (Qiagen, Germany).

Editing efficiency was estimated using the T7 endonuclease I (T7E1) assay, in which heteroduplexes formed by reannealed PCR products were digested at mismatched sites. Band intensities were quantified using ImageJ software. Mutation verification was performed by Sanger sequencing, and chromatograms were analyzed using SnapGene software.

### 3.7. Quantification of CDKL5 mRNA Expression

Total RNA was extracted from wild-type and edited cells using the Thermo Fisher RNA Purification Mini Kit, and cDNA was synthesized from 1 μg RNA using the First-Strand cDNA Synthesis Kit (Thermo Fisher). Quantitative PCR was performed using SYBR Green Master Mix on a Bio-Rad CFX96 system with the following primers:

CDKL5 forward: 5′-GACATCTGGAGTTCGAGTGG-3′

CDKL5 reverse: 5′-GAGGTCATAGGACACACCTG-3′

GAPDH forward: 5′-GAAGGTGAAGGTCGGAGTC-3′

GAPDH reverse: 5′-GAAGATGGTGATGGGATTTC-3′

Relative gene expression was calculated using the 2^-ΔΔCt^ method and normalized to GAPDH. All experiments were performed in triplicate.

### 3.8. Cell Viability and Apoptosis Assays

Cell viability was assessed using the MTT assay. Cells were seeded in 96-well plates at 1 × 10^4^ cells/well. After 48 hours, 20 μL of 5 mg/mL MTT was added to each well. Formazan crystals were solubilized in 150 μL dimethyl sulfoxide (DMSO), and absorbance was measured at 570 nm. Results were expressed as a percentage relative to wild-type controls.

Apoptosis was quantified using Annexin V-fluorescein isothiocyanate (FITC)/PI staining followed by flow cytometry (BD Accuri C6). Early and late apoptotic cells were expressed as a percentage of total live cells. Caspase-3 activity was measured using a colorimetric assay kit (Abcam, ab39401) at 405 nm and normalized to total protein content.

### 3.9. Western Blot Analysis

Total protein was extracted from HEK293T cells using ice-cold radioimmunoprecipitation assay lysis buffer (50 mM Tris-HCl, pH 7.4; 150 mM NaCl; 1% NP-40; 0.5% sodium deoxycholate; and 0.1% sodium dodecyl sulfate [SDS]) supplemented with a protease and phosphatase inhibitor cocktail (Roche). Protein concentration was determined using the Bradford assay (Bio-Rad). Equal amounts of protein (20 μg) were separated by 10% SDS-polyacrylamide gel electrophoresis and transferred onto 0.45-μm nitrocellulose membranes (Bio-Rad) using a semidry transfer system.

Membranes were blocked with 5% non-fat dry milk in Tris-buffered saline containing 0.1% Tween-20 for 1 hour at room temperature, followed by overnight incubation at 4°C with primary antibodies: rabbit anti-CDKL5 (Abcam, ab191229; 1:1000) and mouse anti-GAPDH (Abcam, ab9485; 1:5000) as a loading control. After 3 washes with Tris-buffered saline containing Tween-20, membranes were incubated with horseradish peroxidase-conjugated secondary antibodies (anti-rabbit or anti-mouse immunoglobulin G; 1:5000) for 1 hour at room temperature. Protein bands were visualized using enhanced chemiluminescence substrate (Clarity Western ECL Substrate, Bio-Rad) and detected with a ChemiDoc MP Imaging System (Bio-Rad). Band intensities were quantified using Image Lab software (version 6.1, Bio-Rad). CDKL5 mRNA expression levels were normalized to GAPDH and expressed relative to wild-type controls. All experiments were performed in biological triplicate.

### 3.10. Statistical Analysis

Statistical analyses were performed using GraphPad Prism 9.0 (GraphPad Software, USA). All experiments were performed in triplicate (n = 3), and results are presented as mean ± standard deviation (SD). Statistical significance between groups was determined using unpaired 2-tailed Student t tests or 1-way analysis of variance with Bonferroni post hoc correction. A P value < 0.05 was considered statistically significant.

## 4. Results

### 4.1. Validation of sgRNA Design and CRISPR/Cas9 Vector Construction

**Figure 1.**
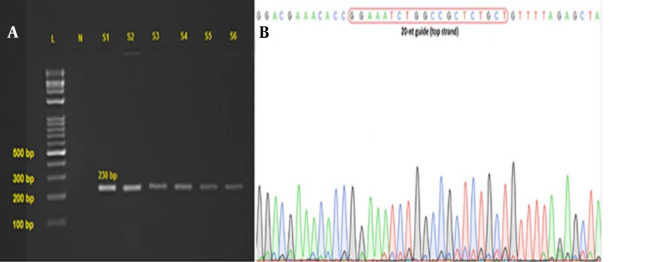
Validation of sgRNA insertion into the PX458 vector. (A) Colony PCR analysis showing the expected approximately 230-bp amplicon in representative transformed colonies. L, DNA ladder; N, negative control; S1-S6, representative positive colonies. Overall, 23 of 25 colonies were positive, indicating a cloning efficiency of 92%. (B) Sanger sequencing chromatogram confirming correct sgRNA insertion into the PX458 vector; the boxed region represents the 20-nt sgRNA target sequence. Results are representative of 3 independent biological replicates.

The sgRNA targeting exon 2 of CDKL5 (5′-GGAGTTTGTCTTCATGAAGA-3′) was selected based on its high predicted on-target score of 0.84 and minimal off-target probability (< 0.1%). The sgRNA sequence was successfully cloned into the PX458 plasmid vector using BbsI restriction enzyme digestion and T4 ligation. Colony PCR screening of *E. coli* DH5α transformants confirmed correct sgRNA insertion in 92% (23 of 25) of colonies, and sequencing verified accurate integration without frameshift errors (Figure 1).

The final construct, pSpCas9(BB)-2A-GFP-sgRNA(CDKL5), expressed GFP under the CAG promoter, enabling visual confirmation of transfection efficiency in mammalian cells.

### 4.2. Transfection Efficiency and GFP-Based Sorting

**Figure 2.**
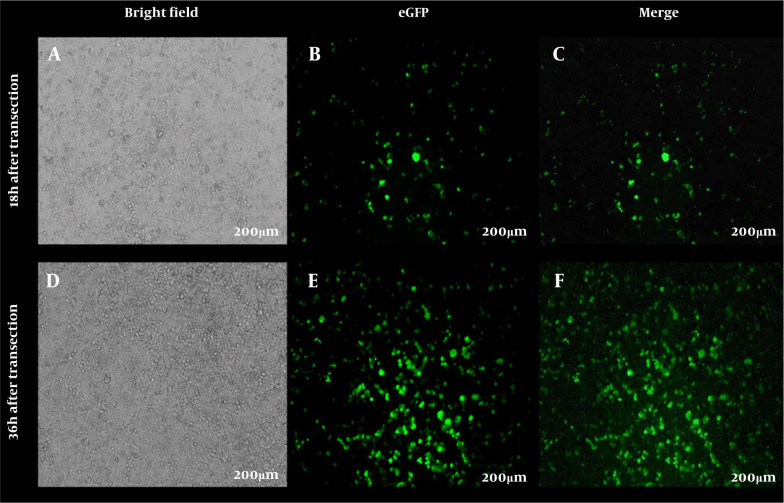
Temporal analysis of GFP expression in HEK293T cells following CRISPR/Cas9-mediated transfection. A-C, Representative bright-field (A), eGFP fluorescence (B), and merged (C) images of HEK293T cells at 18 hours after transfection. (D-F) Representative bright-field (D), eGFP fluorescence (E), and merged (F) images of HEK293T cells at 36 hours after transfection. Scale bars: 200 μm. All images are representative of 3 independent biological replicates (n = 3).

HEK293T cells transfected with the CRISPR/Cas9 construct exhibited strong GFP fluorescence 48 hours after transfection (Figure 2). Flow cytometry quantification indicated an average transfection efficiency of 41.6 ± 3.2% (n = 3). Fluorescence-activated cell sorting yielded approximately 3.9 × 10^4^ GFP-positive cells per well, which were expanded for genotyping. Cell morphology and growth remained normal after sorting, suggesting no cytotoxic effects from transient Cas9 expression (Figure 2).

### 4.3. Analysis of Genome Editing Efficiency

**Figure 3.**
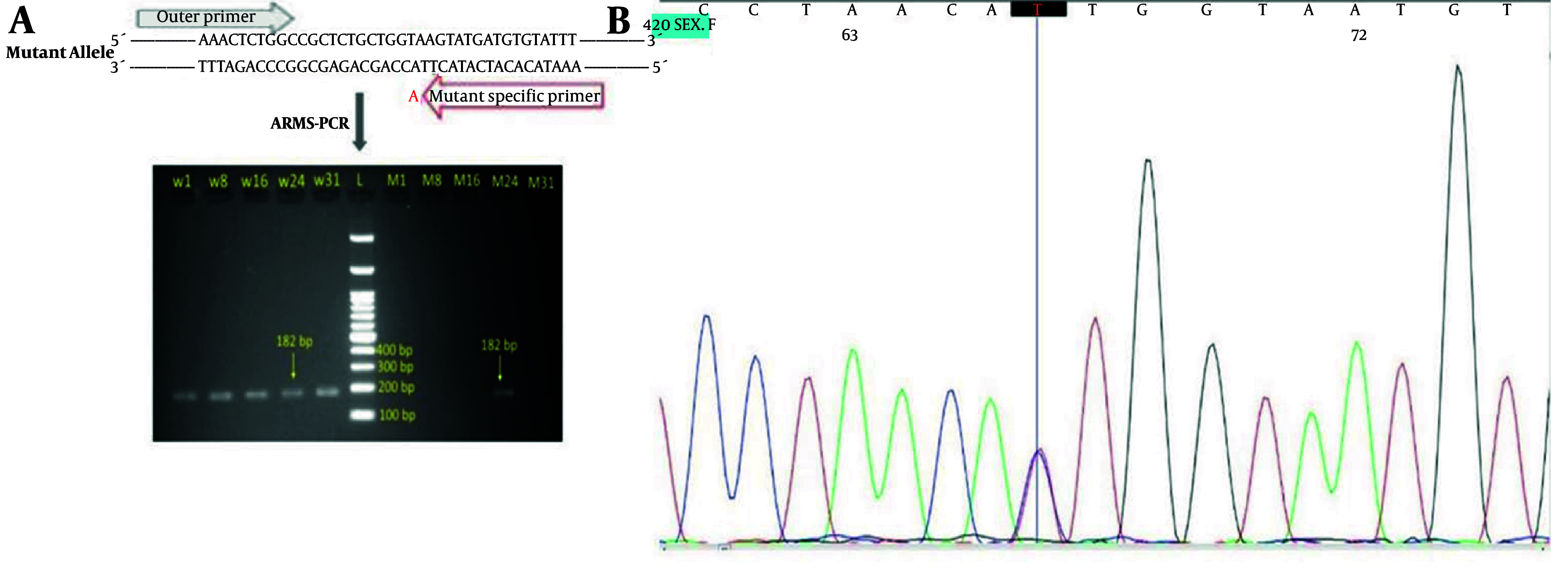
Validation of HDR-edited CDKL5 mutant clones. A, ARMS-PCR screening of the CDKL5 exon 2 region in expanded clones. For each clone, 2 separate allele-specific reactions were performed. Lane L: 100-bp DNA ladder; lanes w1, w8, w16, w24, and w31: ARMS-PCR reactions using the wild-type-specific primer; lanes M1, M8, M16, M24, and M31: ARMS-PCR reactions using the mutant-specific primer. Both wild-type and mutant alleles produced a 182-bp amplicon when their respective primers were used. Clone 24 generated 182-bp bands in both the wild-type-specific reaction (w24) and the mutant-specific reaction (M24), indicating the presence of both wild-type and c.172A>T mutant alleles and confirming successful heterozygous HDR-mediated editing. B, Representative Sanger sequencing chromatogram verifying the c.172A>T substitution, which results in the p.Lys58Met amino acid change. Data are representative of 3 independent biological replicates (n = 3).

After expansion of sorted GFP-positive cells, individual clones were screened by ARMS-PCR to identify candidate edited clones. ARMS-PCR amplification of the CDKL5 exon 2 region showed that clone 24 contained positive bands for both mutant and wild-type alleles, indicating that this clone carried the desired mutation in the heterozygous state (Figure 3). In this screening assay, the 182-bp amplicon served as an allele-specific marker; its presence in the wild-type-specific reaction confirmed retention of the native sequence, whereas its presence in the mutant-specific reaction indicated successful HDR-mediated c.172A>T substitution. Simultaneous detection of the 182-bp product in both reactions for clone 24 (lanes w24 and M24) provided initial confirmation of its heterozygous genotype. To further validate and categorize editing outcomes, a subset of randomly selected clones was analyzed by Sanger sequencing.

Sanger sequencing of randomly selected clones (n = 20) showed that 28% carried heterozygous indel mutations, 12% exhibited HDR-mediated single-nucleotide substitutions (c.172A>T), and 60% retained the wild-type sequence (Figure 3). HDR-specific clones showed a clean A-to-T substitution resulting in a lysine-to-methionine change at residue 58 (p.Lys58Met). No insertions larger than 10 bp were observed, suggesting efficient repair and low off-target Cas9 activity.

### 4.4. Reduction of CDKL5 mRNA Expression in Edited Cells

**Figure 4.**
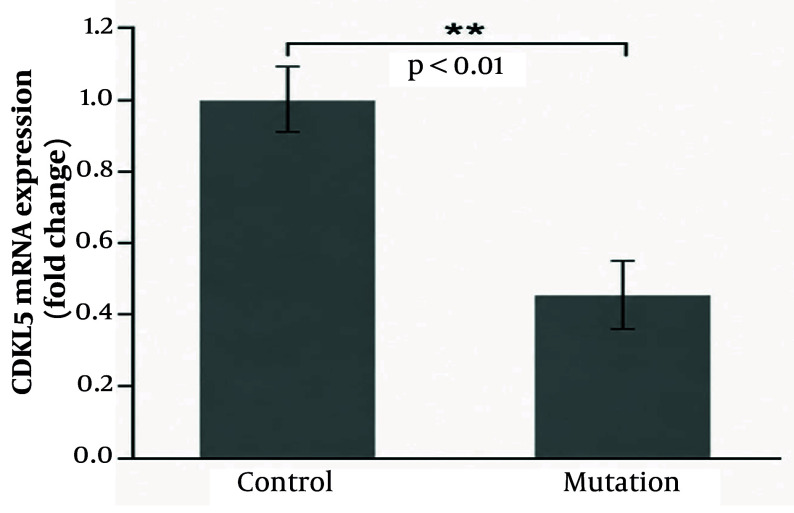
Impact of the c.172A>T mutation on CDKL5 mRNA expression. Relative CDKL5 mRNA levels were quantified by RT-qPCR and normalized to GAPDH. The mutation significantly reduced CDKL5 mRNA expression to approximately 0.45-fold compared with the control. Data are presented as mean ± SD (n = 3). Statistical significance was determined using an unpaired 2-tailed Student *t*-test. **P < 0.01.

To determine the effect of the introduced mutation on gene expression, CDKL5 mRNA levels were quantified by RT-qPCR in wild-type and edited clones (n = 3). As shown in Figure 4, CDKL5 transcript levels were significantly reduced in mutant cells, reaching 0.45 ± 0.08-fold relative to wild-type cells (P < 0.01). The absence of a significant difference in GAPDH expression confirmed stable normalization. These results suggest that the p.Lys58Met substitution may destabilize CDKL5 mRNA and/or interfere with transcriptional regulation.

### 4.5. Reduced Viability and Enhanced Apoptosis

Cell viability, measured using the MTT assay, showed a 25 ± 3% decrease in metabolic activity in mutant cells compared with wild-type controls (P < 0.05). This decline was consistent across all HDR-positive clones, suggesting a mutation-dependent phenotype rather than nonspecific transfection toxicity.

Flow cytometric analysis of Annexin V-FITC/PI staining revealed a significant increase in apoptotic cell populations. The proportion of early apoptotic cells increased from 6.8 ± 1.1% in wild-type cells to 15.7 ± 1.9% in mutant clones, and late apoptotic/necrotic cells increased from 4.1 ± 0.9% to 11.3 ± 1.6% (P < 0.01).

**Figure 5.**
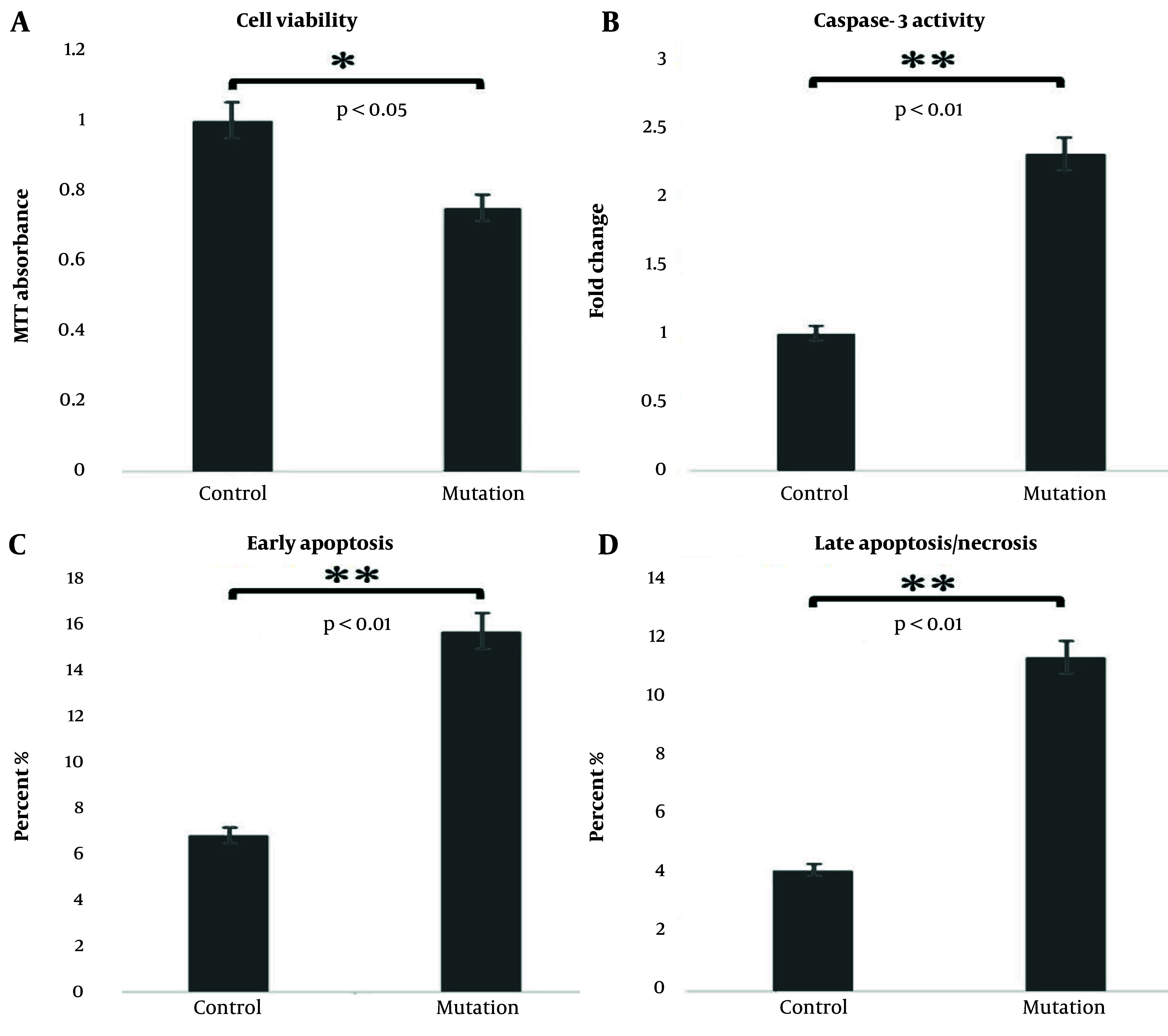
Analysis of cell viability and apoptosis in CDKL5 mutant cells. A, Cell viability measured by MTT assay; B, relative caspase-3 enzymatic activity; C, Percentage of early apoptotic cells; and D, late apoptotic/necrotic cells determined by Annexin V-FITC/PI staining. Data represent mean ± SD from 3 independent biological replicates (n = 3), with each experiment performed in technical triplicate. Statistical significance: * P < 0.05 and ** P < 0.01 compared with wild-type controls.

Caspase-3 enzymatic activity, a hallmark of apoptosis, was elevated 2.3-fold in mutant cells relative to controls (P < 0.01). Collectively, these findings indicate that CDKL5 dysfunction compromises cellular viability, likely through activation of intrinsic apoptotic pathways (Figure 5). A summary of the major quantitative outcomes related to cell viability, apoptosis, necrosis, and caspase-3 activity is provided in Table S1 in the Supplementary File.

### 4.6. CDKL5 Protein Expression Is Significantly Reduced in Mutant Cells

**Figure 6.**
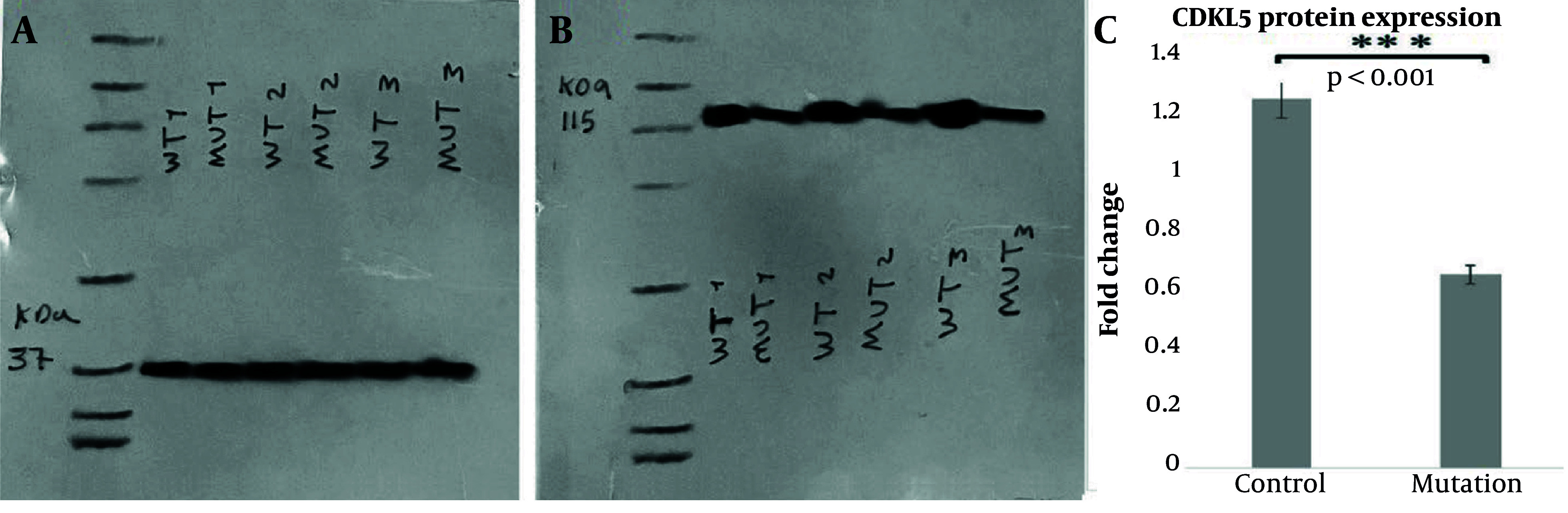
CDKL5 protein expression in wild-type and c.172A>T (p.Lys58Met) mutant HEK293T cells. A, Representative Western blot images showing GAPDH (approximately 37 kDa) as a loading control. B, Representative Western blot images of CDKL5 (approximately 115 kDa) in wild-type (WT) and mutant (MUT) cells across 3 independent biological replicates. C, Densitometric quantification of CDKL5 protein levels normalized to GAPDH. Data are presented as mean ± SD (n = 3). CDKL5 expression was significantly reduced in mutant cells (0.65 ± 0.01) compared with WT controls (1.24 ± 0.03), representing an approximately 48% decrease (*** P < 0.001, unpaired Student *t*-test).

Western blot analysis revealed a substantial reduction in CDKL5 protein levels in mutant cells harboring the c.172A>T (p.Lys58Met) variant compared with wild-type controls. Quantitative densitometry showed that normalized CDKL5 expression (CDKL5/GAPDH ratio) was 0.65 ± 0.01 in mutant samples versus 1.24 ± 0.03 in wild-type samples (P < 0.001, Figure 6), corresponding to a 48% decrease in protein abundance. This reduction in CDKL5 protein expression was consistent across all 3 biological replicates, demonstrating high reproducibility of the phenotype. This pronounced decrease aligns with the RT-qPCR data showing a 55% reduction in CDKL5 mRNA expression, suggesting that the c.172A>T mutation affects transcriptional stability and/or posttranscriptional regulation of CDKL5. The p.Lys58 residue is located within the catalytic kinase domain, and substitution with methionine likely disrupts protein folding, leading to enhanced proteasomal degradation or impaired translation efficiency. These findings provide biochemical evidence that the c.172A>T variant severely compromises CDKL5 expression at the protein level, which may underlie the observed cellular phenotypes, including reduced cell viability and increased apoptosis (Figure 6).

Quantification of CDKL5 protein expression by Western blot analysis is presented in Table S2 in the Supplementary File. Full-length uncropped blots are provided in Figure S1 in the Supplementary File.

## 5. Discussion

In this study, we successfully established a human cellular model of CDKL5-related ASD using CRISPR/Cas9-mediated genome editing. By introducing a specific missense mutation (c.172A>T; p.Lys58Met) within exon 2 of CDKL5, we generated a controlled and reproducible platform for investigating the molecular pathology of CDKL5 deficiency. This model recapitulated key hallmarks of the disorder, including downregulation of CDKL5 expression, compromised cell viability, and increased apoptosis, thereby validating its utility for mechanistic and therapeutic research. The CRISPR/Cas9 system enabled efficient and specific gene editing, with an average on-target activity of 41.6% and an HDR efficiency of 12%. Importantly, no detectable off-target mutations were identified at the top predicted loci, confirming the specificity of the sgRNA design.

These results are consistent with recent reports using CRISPR/Cas9 to manipulate CDKL5 in neuronal and glial cells. Carriero et al. used CRISPR to model CDKL5 deficiency in rat hippocampal neurons, demonstrating impaired synaptic vesicle endocytosis and altered neuronal excitability. Similarly, Halmai et al. used a dCas9-TET1 fusion to epigenetically reactivate CDKL5 on the inactive X chromosome (24). Collectively, these findings and the present results demonstrate that CRISPR systems, whether nuclease-active or catalytically inactive, can recapitulate CDKL5 dysfunction and provide a foundation for therapeutic correction studies.

The observed 55% reduction in CDKL5 mRNA and 48% reduction in protein expression in edited clones suggest that the p.Lys58Met substitution impairs transcript stability and/or translational efficiency. This amino acid lies within the conserved N-terminal serine/threonine kinase domain, a critical region required for substrate phosphorylation and catalytic activity (4, 5). Previous studies have shown that missense mutations in the kinase domain often destabilize the protein or disrupt its subcellular localization (6). Reduced CDKL5 expression has profound implications for neuronal development. In animal models, Cdkl5 knockout leads to dendritic spine immaturity, defective synaptic plasticity, and behavioral deficits reminiscent of ASD (7, 8). The present cellular findings support these observations and confirm that even a single amino acid substitution within the catalytic region can trigger downstream molecular dysfunction.

The increased apoptosis observed in the mutant clones, characterized by a 2.3-fold elevation in caspase-3 activity and a higher proportion of Annexin V-positive cells, corroborates previous evidence that CDKL5 contributes to neuronal survival. Loi et al. demonstrated that Cdkl5-deficient neurons exhibit heightened susceptibility to excitotoxic and oxidative stress, likely due to a defective DNA damage response (25). Similarly, Nicole et al. (2021) reported mitochondrial dysfunction and bioenergetic deficits in neurons derived from CDKL5-mutant iPSC lines (26). In the present study, cell death occurred independently of Cas9 toxicity, as control cells transfected with the empty PX458 vector maintained normal viability. These results suggest that CDKL5 deficiency directly activates apoptotic cascades, possibly through mitochondrial membrane depolarization or impaired phosphorylation of neuroprotective targets, such as MeCP2 and HDAC4 (11, 12). Indeed, CDKL5-mediated phosphorylation of MeCP2 is essential for chromatin regulation and synaptic maturation, and disruption of this pathway may account for overlapping phenotypes between CDKL5 deficiency and Rett syndrome (13).

Several previous studies have used patient-derived fibroblasts or iPSCs to study CDKL5 deficiency; however, such systems often exhibit high genetic variability. For instance, Nicole et al. (2021) showed that CDKL5 mutations in iPSC-derived neurons cause mitochondrial transport defects and abnormal calcium homeostasis (26). However, these lines inherently contain patient-specific polymorphisms, making it difficult to distinguish primary disease-causing effects from secondary, background-related effects. The generation of a CDKL5-mutant human cell model provides an important platform for understanding the molecular relationship between ASD and epileptic encephalopathies. CDKL5 interacts with numerous ASD-related genes, including SHANK3, NRXN1, and MECP2, converging on pathways involved in synaptic regulation and chromatin remodeling (15). Disruption of these networks contributes to an abnormal excitatory/inhibitory balance in cortical circuits, a hallmark of autism (16, 17).

CDKL5 phosphorylates key synaptic proteins, including MeCP2 at Ser421 and MAP1S, thereby modulating chromatin architecture and microtubule stability, respectively. The p.Lys58Met mutation, located in the ATP-binding pocket of the kinase domain, is predicted to abolish catalytic activity, leading to hypophosphorylation of these substrates. This aligns with the observed reduction in cell viability and increase in apoptosis, phenotypes also reported in SHANK3 R1117X knock-in models and SCN2A L1342P iPSC-derived neurons, in which single-nucleotide substitutions similarly induced caspase-3 activation and metabolic stress without overt neuronal differentiation (27).

Although CDD is primarily associated with impaired neuronal development and function, HEK293T cells were intentionally selected in this study as an initial human cellular platform for CRISPR/Cas9-mediated knock-in generation rather than as a complete neuronal disease model. HEK293T cells offer several technical advantages for precise genome editing, including high transfection efficiency, rapid and robust proliferation, reproducible culture conditions, and efficient clonal expansion and screening after editing. These features are particularly important for knock-in strategies, in which HDR-mediated precise editing is technically challenging and often requires extensive screening and validation of edited clones. In this context, the HEK293T-based system enabled reliable introduction and validation of the pathogenic CDKL5 c.172A>T variant and allowed assessment of its immediate molecular and cellular consequences, including reduced CDKL5 expression, decreased cell viability, increased apoptosis, and evaluation of predicted off-target loci.

However, the non-neuronal origin of HEK293T cells limits their ability to model neuron-specific aspects of CDD, such as synaptic maturation, dendritic development, calcium signaling, and electrophysiological activity. Therefore, this model should be interpreted as an initial molecular validation platform, and future studies using iPSC-derived neurons, neuronal precursor cells, or other disease-relevant neuronal systems will be necessary to extend these findings in a more physiologically relevant context. While this study demonstrates the successful generation of a CDKL5-mutant cellular model, certain limitations should be acknowledged. First, HEK293T cells are non-neuronal and may not fully capture the neuron-specific regulatory dynamics of CDKL5. Future studies using neuronal precursor cells or iPSC-derived neurons would provide improved physiological relevance. Second, the analysis focused on gene expression and apoptosis; however, additional functional assays, such as electrophysiological recordings, calcium imaging, or synaptic-marker quantification, could further elucidate the impact of CDKL5 loss on neuronal connectivity. Finally, although off-target analysis revealed no significant mutations in the top 5 predicted sites, comprehensive genome-wide off-target mapping using GUIDE-seq or whole-genome sequencing would enhance confidence in the genetic precision of the model.

### 5.1. Conclusions

This study successfully established a human cellular model of ASD associated with CDKL5 deficiency using the CRISPR/Cas9 genome-editing system. By introducing a defined missense mutation (c.172A>T; p.Lys58Met) into exon 2 of CDKL5, we achieved precise genomic modification with high specificity and reproducibility. The resulting mutant cells displayed markedly reduced CDKL5 mRNA and protein levels, decreased cell viability, and significantly increased apoptosis. These findings validate CDKL5 as a central regulator of cellular survival pathways and highlight its essential role in neuronal integrity. This model provides a useful in vitro platform for exploring the molecular underpinnings of CDKL5-related neurodevelopmental disorders and testing emerging therapeutic strategies, such as CRISPR-based correction, epigenetic reactivation, and small-molecule modulation. Although the HEK293T-based model offers a useful and reproducible platform for preliminary mechanistic studies and therapeutic screening, its non-neuronal origin limits its ability to fully recapitulate the neuronal context of CDD. Accordingly, future studies using iPSC-derived neuronal models or neuronal cell lines will be necessary to validate and extend these findings in more physiologically relevant contexts.

ijpr-25-1-172311-s001.pdf

## Data Availability

The data presented in this study are uploaded during submission as a supplementary file and are openly available for readers upon request.
