# Supplementary material for: CRISPR/Cas9-Engineered HEK293T Cellular Model Harboring the Pathogenic CDKL5 c.172A>T Variant Recapitulates Core Molecular Phenotypes of CDKL5 Deficiency Disorder
Source: Iran J Pharm Res. 2026 Jun 13;25(1):e172311. doi: 10.5812/ijpr-172311 (PMC13389376; doi:10.5812/ijpr-172311)
Supplement: ijpr-25-1-172311-s001.pdf [file ijpr-25-1-172311-s001.pdf]

Table S1: a concise overview of the major quantitative outcomes related to apoptosis and necrosis observed in this study.

**Parameter**	**Wild-type**	**Mutant (CRISPR-Edited)**	**% Change**	**p-value**
Cell viability (MTT absorbance)	1.00 ± 0.04	0.75 ± 0.05	−25%	0.015
Early apoptosis	6.8 ± 1.1%	15.7 ± 1.9%	+131%	0.004
Late apoptosis/necrosis	4.1 ± 0.9%	11.3 ± 1.6%	+175%	0.003
Caspase-3 activity (fold-change)	1.00 ± 0.12	2.30 ± 0.19	+130%	0.002

Table S2. Quantification of CDKL5 protein expression by Western blot analysis

**Sample**	**Replicate**	**CDKL5 (AU)**	**GAPDH (AU)**	**Normalized CDKL5 (CDKL5/GAPDH)**
Wild-type (WT)	1	125.4	98.5	1.27
Wild-type (WT)	2	118.9	97.8	1.22
Wild-type (WT)	3	122.5	99.2	1.24
**WT Mean ± SD**	—	—	—	**1.24 ± 0.03**
Mutant (MUT)	1	62.3	97.9	0.64
Mutant (MUT)	2	65.1	98.4	0.66
Mutant (MUT)	3	63.8	99.0	0.64
**MUT Mean ± SD**	—	—	—	**0.65 ± 0.01**
**Fold change (MUT/WT)**	—	—	—	**0.52**

- AU: Arbitrary units; SD: Standard deviation.
- Normalized CDKL5 values were calculated as the ratio of CDKL5 signal to GAPDH signal. Fold change represents the ratio of MUT mean to WT mean. A fold change value of **0.52** corresponds to a **~48% decrease** in protein expression in the Mutant group compared to the Wild-type.

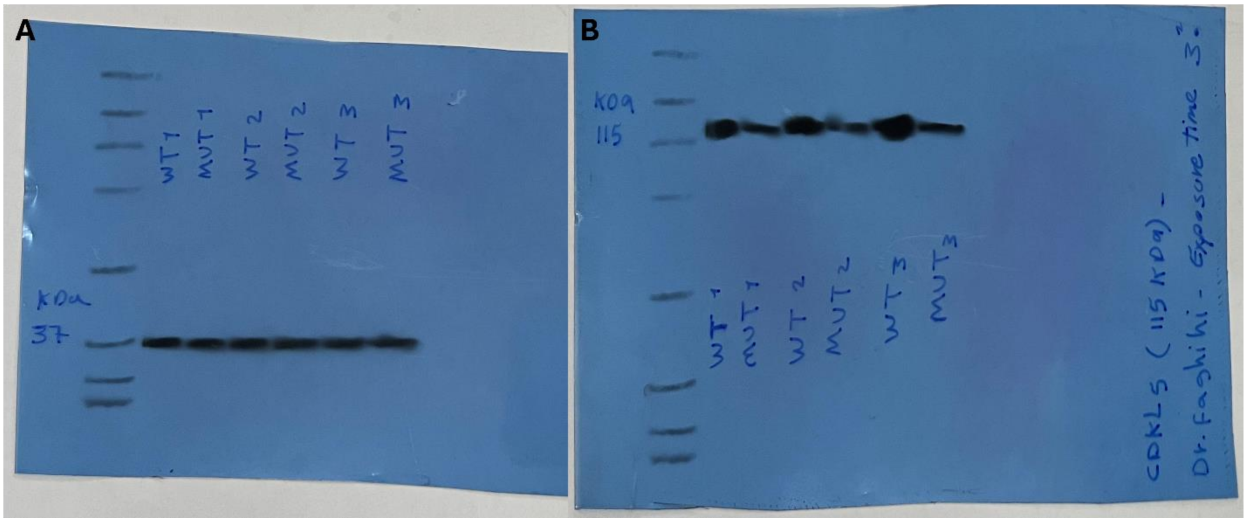

Supplementary Figure S1. Full-length uncropped Western blot membranes for GAPDH and CDKL5.

(A) Uncropped Western blot membrane for GAPDH (~37 kDa) used as the loading control. The molecular weight marker (ladder) is shown on the left, with the 37 kDa band explicitly labeled. Lanes correspond to three biological replicates of wild-type (WT 1–3) and mutant (MUT 1–3) HEK293T cells. (B) Uncropped Western blot membrane for CDKL5 (~115 kDa). The protein ladder on the left indicates the molecular weight markers, with the target band at 115 kDa. The significant reduction in CDKL5 protein expression in mutant (MUT) samples compared to wild-type (WT) is clearly visible. Original experimental notations, including the protein name (CDKL5), molecular weight (115 kDa), and exposure time, are preserved on the right side of the membrane to ensure full transparency and data integrity.
